# Social support needs of first-time parents in the early-postpartum period: A qualitative study

**DOI:** 10.3389/fpsyt.2022.1043990

**Published:** 2022-12-14

**Authors:** Elisabeth Schobinger, Mélanie Vanetti, Anne-Sylvie Ramelet, Antje Horsch

**Affiliations:** ^1^Institute of Higher Education and Research in Healthcare (IUFRS), University of Lausanne, Lausanne, Switzerland; ^2^Department Woman-Mother-Child, University Hospital, Lausanne, Switzerland

**Keywords:** social support, early postpartum, mothers, fathers, needs, qualitative

## Abstract

**Background:**

The early postpartum period is a critical time for first-time parents as they adapt to their new role. Perceived lack of social support is a risk factor for developing mental health problems. Insufficient or inappropriate professional support for both parents has been reported by many studies. Social support that appropriately meets parents' needs is an important protective factor for parents' wellbeing; however, little is known about the social support needs of both first-time parents.

**Aims and objectives:**

To describe both first-time parents' formal social support needs in the early postpartum period.

**Method:**

Individual semi-structured interviews were conducted with first-time parents recruited on the postpartum ward of a Swiss university hospital. Thematic analysis was used to identify themes and sub-themes.

**Results:**

Fifteen mothers and eleven fathers were interviewed. Twelve themes were identified. Mothers' themes were “experiencing postpartum changes,” “creation of a family unit,” “self-esteem,” “emotional needs,” “difficulty in communicating their needs,” and “the postpartum stay.” Fathers' themes were “to be included in care procedures on the postpartum ward,” “to be reassured,” “to anticipate their postpartum stay” and “to consider their need as non-priority.” Parental shared needs were: “to care for their newborn,” and “returning home.”

**Conclusion:**

Mothers' and fathers' needs differed. Mothers needed more emotional support than fathers and fathers considered themselves as the main support for their partner. Fathers wanted to be integrated in the care of their newborn.

## Introduction

Transition to parenthood is a challenging time, especially for first time mothers and fathers (1–3) who have to adjust to their new role, and the emotional and social changes (4, 5). The early postpartum period (from 2 to 7 days after birth) (6) is a critical time, because it can lead to different mental health problems, known under the term perinatal mood and anxiety disorders (PMAD), such as depression and childbirth-related posttraumatic stress disorder (CB-PTSD) (7–10). Mental health complications related to childbirth can impact the whole family, with dramatic consequences for both parents and the newborn (i.e., difficulty in establishing the mother-infant bond and in initiating breastfeeding, child's sleep and development impairments, and difficulties in couple relationship) (10–16).

Risk factors contributing to maternal and paternal PMAD, such as CB-PTSD and/or depression include a history of mental illness, being primipara, operative birth/having complications during birth, negative subjective experience of birth (10, 17–19), poor coping strategies and perceived lack of social support during and after birth (12, 17, 18, 20).

Social support is a reciprocal exchange of resources or activities between at least two individuals, aiming to improve the health and wellbeing of the person receiving it. There are two sources of support: formal [healthcare providers (HCPs)] and informal (friends, family), although most studies so far have not clearly distinguished these two types (21–23). Both formal and informal social support are protective factors in the postpartum period (24, 25). On the one hand, when perceived as sufficient, social support moderates stress in parents and is negatively correlated with postpartum anxiety/depression and CB-PTSD symptoms (26–28). Social support facilitates bonding with the child (2, 23), and increases parental self-efficacy and sense of security (2, 23, 29, 30). On the other hand, lack of social support is negatively associated with emergency visits after discharge. The lower the level of support, the higher was the risk of an emergency visit (31). Mothers not satisfied with their relationship with HCPs at birth, had a six times higher risk to report a negative birth experience (32). Lack of support in the postpartum period contributed to paternal negative emotions and psychological distress (33, 34).

Evidence shows that parents are often not satisfied with the formal social support they received in postpartum care (9, 35, 36), because their needs were unmet or not addressed adequately (37–41). While mothers want HCPs to involve fathers more in care (42), a majority of fathers (63%) report to be unsatisfied with their participation in care (43).

In summary, formal social support is important in the postpartum period, especially for first-time parents. Given the lack of distinction of formal and informal social support in previous studies, further research is needed to better understand both mothers' and fathers' formal social support needs during this particularly vulnerable period (34). This may help HCPs to better tailor their support to the individual needs of first-time parents and thus inform early postpartum care practices.

The objective of this study was to describe first-time parents' perceived formal social support needs in the early postpartum period.

## Materials and methods

### Design

A qualitative descriptive study design using a phenomenological approach was used, as it is most suited to describing personal experiences of an event or situation (44). This approach belongs to the constructivism paradigm, which assumes that knowledge is maximized when the distance between researcher and participants is reduced (45).

### Participant recruitment

Participants were recruited on two postpartum wards (one with single rooms and one with double rooms) of a Swiss University Hospital from June to September 2021. Purposive sampling was used, based on the following inclusion criteria: (a) primiparous women (over 18 years of age), and who gave birth to a healthy full-term newborn (≥37 gestational weeks) by cesarean section or vaginal birth; and/or (b) their partner who is parent for the first time, over 18 years of age, was present during birth, and stayed on the postpartum ward. Exclusion criteria for both parents included insufficient knowledge of French, child's admission to the Neonatal Intensive Care Unit (NICU), stillbirth, or death of their child within the 24 h post-delivery. Due to information power and methodological guidance, we planned to recruit ~15 mothers and 15 fathers (46, 47). Parents were individually invited, by the researcher, to take part in the study from the 2nd day after birth to hospital discharge (which is usually on day 4). Consenting parents were contacted by phone, and a date/time/modality (online or at the parent's house) was agreed for the interview to take place. All interviews were held within the first 2 weeks after birth and after discharge.

### Data collection

Data collection involved individual semi-structured interviews in French, conducted from June to October 2021 by a trained registered nurse and PhD candidate in nursing sciences at the time of data collection. Due to COVID-19 restrictions, parents were offered the choice of being interviewed either at home or ***via*** a secure online “face-to-face” platform (ZOOM). Interviews were held online for 57% of participants. Interviews were recorded using a separate digital recording device to safeguard confidentiality and subsequently transcribed verbatim. As little is known about formal social support needs of first-time parents in the early postpartum period, an interview guide with open-ended questions (Table 1) was developed to ensure relative consistency across interviews. This guide was developed with experts from the field and pilot-tested with one couple. No questions were asked about the birth experience, as the focus of the study was on the early postpartum period. A coded questionnaire to collect sociodemographic information was completed at the end of the interview by participants.

**Table 1:** Interview guide.

Introduction	Welcome and introduction Acknowledgment for participating in the study Explanation about recording and transcribing
Social support needs of first-time parents	Once you or your partner had given birth when you arrived at the postpartum ward, what were your social support needs? What are the things you would have wanted to be done for you? Words that you wanted to be said? How you would have liked to be treated or your baby or your partner?
Conclusion	Feedback of the main points to the participants Collection of sociodemographic data and acknowledgment of their time.

### Data analysis

Descriptive statistics were used to describe the sample and determine the mean interview time. Inductive thematic analysis was used to analyse the data (48). Interviews were coded independently by two researchers (ES, nurse and MV, sociologist). Codes were compared and agreed upon, and then discussed with the PhD supervisors (AH, clinical psychologist and ASR, pediatric clinical nurse specialist). This process was also used for generating the themes and to ensure the credibility of the results. Analysis was conducted with MAXQDA 2020^©^. Results were presented to two randomly selected mothers who had participated in the study to ensure the validity of the findings. No fathers participated during this process, despite being invited.

### Ethical considerations

This study was approved by the ethics committee for research on humans of the Canton of Vaud, Switzerland (Project No 2021-00762). Written informed consent was obtained from all participants. Transcripts were anonymised prior to data analysis. All audio-recordings were destroyed immediately after transcription. In order to protect participants' confidentiality, fictional codes were used to represent mothers and fathers quotes in the article. For example, F7 in the quotes represents participant seven of the fathers.

## Results

### Participant characteristics and study setting

Fifteen mothers and eleven partners who identified themselves as fathers participated, including eleven couples and four mothers participating alone. No same-sex couple took part in this study. All participants were employed (on maternity or paternity leave) when participating in the study. Every participant had a health insurance as it is mandatory in Switzerland. Regarding the participants' origin, the majority were Swiss. European participants were from Western Europe. Non-European participants were from Brazil, Indonesia and Senegal. Table 2 presents the sample characteristics. The median recorded interview time was 45 min (ranging from 33 to 75 min). In the hospital where the study took place, no patients or partners are routinely screened for PMAD. Every participant kept their newborn in their room during their postpartum stay, as rooming-in was routine-practice. No participant received support from a lactation counselor. Partners were allowed in double room from 8 am to 10 pm and without any restriction in single room. Partners had no restrictions due to COVID-19. Furthermore, every parent was given documentation about newborn's care. These documents contained information on community-based resources, such as community midwife and about the availability of the early childhood nurses.

**Table 2:** Sample characteristics.

	**Frequency (%)**	**Mean (SD)**
Age (years; mean, SD)		35 (3.3)
**Nationality (** ***n*** **, %)**		
Swiss	15 (57.7%)	
Other European	8 (30.8%)	
Non-European	3 (11.5 %)	
**Origin culture (** ***n*** **, %)**		
Europe	21 (80.8%)	
Latin America	1 (3.9%)	
Africa	2 (7.7%)	
Asia	2 (7.7%)	
**Marital status (** ***n*** **, %)**		
Co-habiting/Married	26 (100%)	
**Mode of birth**		
Vaginal birth	16 (61.5%)	
Instrumental vaginal birth	4 (15.4%)	
Planned cesarean section	4 (15.4%)	
Unplanned cesarean section	2 (7.7%)	
**Moment of birth (weeks of gestation)**		
≥37 and < 39 weeks	10 (38.5%)	
≥39 to <41weeks	7 (26.9%)	
Over 41 weeks	9 (34.6%)	
Birth weight (g, mean, SD)		3,298 (447)

### Themes

In total, 12 themes were identified and classified into three categories: mothers' needs (*n* = 6): “experiencing postpartum changes,” “emotional needs,” “creating a family unit,” “self-esteem,” “difficulty in communicating their needs” and “postpartum stay,” fathers' needs (*n* = 4): “to be integrated during the postpartum stay,” “to be reassured,” “to anticipate their postpartum stay” and “to consider themselves as non-priority,” and shared parental needs (*n* = 2): “to care for their newborn” and “returning home.” Table 3 presents the themes, and sub-themes (*N* = 29) with illustrative verbatims.

**Table 3:** Themes and sub-themes.

**Categories**	**Themes**	**Sub-themes**	**Verbatims**
3.2.1 Mothers' needs	3.2.1.1 Experiencing postpartum changes	Breastfeeding	“*I was one of the first (needs), I think, it was really important to me. To be able to breastfeed. And I knew from what I read or heard from friends, that the beginnings are important*.” (M6)
		Pain	“*Well, I found it really hard to have to take care of this little being who depends on us in his physical state, and for me, that was the difficulty. I had just been through hell with the birth. And then I was, I was still in a lot of pain, I had difficulty moving and... having to look after the little one in that state was really super hard.”* (M11)
		Sleep deprivation	“*I was crying in the hallway with my baby in my arms I was at the end of my tether. I was exhausted. I hadn't slept for 48 h. And she (the baby) was crying because she was hungry. And I had to insist to make them take my child so I could sleep*.” (M10)
		Postpartum state of mind	“*Especially in the first few hours, I was still really stoned from the c-section and the epidural, so I was really high. [...] He was so calm that I was just like “oh yeah, I've got a baby. That's great.”*” (M12)
	3.2.1.2 Emotions	To be reassured	“*Maybe to have reassuring words such as “it's normal that she's crying, it's going to be ok.” It's always good to have reassuring words.”* (M15)
		To be emotionally supported	“*I also wanted to be mothered, in this context, yes to be mothered. That someone took care of me*.” (M14)
	3.2.1.3 Creating a family unit	Partner's presence	“*The presence of the father for me was crucial*.” (M11)
		Need of intimacy	“*For me, it is essential that we (mothers) should all have single rooms. This moment is so personal we should be alone*.” (M12)
	3.2.1.4 Self-esteem	To do things by myself	“*To be all the time with your baby it's helpful. The first night, I would have appreciated that they took the baby at the nursery, for my own comfort, to rest. But in the same time to be thrown in at the deep end, it's our new reality now. You have to go with it!”* (M17)
		To share with peers	“*It would be interesting to have a place where people can meet, like a kind of small sitting room. It could be an idea*.” (M7)
	3.2.1.5 Difficulty in communicating their needs	Personality	“*At the beginning I couldn't stop apologizing for disturbing them… They told me I do not need to apologize*.” (M12)
		Expectations toward healthcare professionals	“*I thought I should not need to ask but… maybe I should have asked*.” (M1)
	3.2.1.6 Postpartum stay	Continuity of care	“*But it's true that, depending on the person, it can also be quite destabilizing, in fact, to have different opinions, when in fact you don't know what to do. We don't know what is normal and what we want to do as well as possible [...] in fact I think what I want to say is that we would have liked to have had a midwife to follow us throughout.”* (M7)
		Coherent information	“*I had the impression of a lot of contradictory information […]. Somebody told me “the ombilical cord should be out of the diaper.” and then the next day another one told me: “No it should be in the diaper.” I felt lost*.” (M3)
3.2.2 Fathers' needs	3.2.2.1 To be integrated during the postpartum stay	To be included in care procedures	“*After the birth, it's true that it's very exhausting, you have a lot of emotions coming up, etc. And you don't necessarily think to ask. And we don't necessarily think to ask. I think it's something we should have asked for. […] We didn't think about it at the time, but it would have been nice to have at least a little something, not necessarily a real meal, but at least a little something to... To eat*”. (F15)
		Sleep deprivation	”*I think that a bed... would be more suitable. But at the same time, we're not going to die because we spend a week on an air mattress, eh*?” (F13)
		Postpartum state of mind	“*I was very emotional… which means that... (laughs) everything passes through the filters. We don't capture everything 100%.”* (F5)
		To stay with their partner	“*From the beginning, I try to be present and evidently for me it was important to have the possibility to be there for the first bath, diaper […] To be there, to listen and to be useful if I can*.” (F17)
	3.2.2.2 To be reassured	Mother's and newborn's health	“*I was able to join her with the little one, but I went just to see her for ten minutes basically, to see that everything was okay, that she was okay. Also to calm down because obviously I didn't know how things were going for her*.”(F11)
	3.2.2.3 To be prepared for the postpartum stay	To picture themselves in the future	“*My main concern was to know how the postpartum stay will unfold*.” (F10)
		To have point of reference	“*Do they do the first change or is it us? Will they help us or not? For the bath: will they do it? Is it mandatory?*” (F1)
	3.2.2.4 To consider their needs as non-priority	To consider themselves as non-priority	“*Well I don't know how to put it, it' I have the impression that I didn't show myself, um... the need for someone to come and support me emotionally, […]my most important emotional need, I think, was to feel that my partner was supported at times when I wasn't there*.” (F6)
		To be their partner's support	“*The main element of the play is the mother who gives birth, to support her, so I consider myself as a supporting element too.”* (F2)
		Expectation regarding the hospital	“*It's reassuring. I find that this is what most parents are looking for, [...] to have people available in case of a problem, and to be quickly on the spot. knowing that at the (hospital), well we know that.... That at the level of the birth itself, we know that if there is the slightest problem, well that we, at least in (hospital), are dealing with specialists*.” (F14)
		Personality	“I*... didn't want to disturb them either. I thought that when I rang the bell, they might be busy […] I also tend to... try to wait as long as possible when I really need something*.” (F1)
3.2.3 Shared parental needs	3.2.3.1 To care for their newborn	To be supported to learn to care for a newborn	“*The needs we had… it was related to… having help and support and advices because it was our first child. We discovered everything*.” (F15)
		Newborn complications	“*It (tremors)gives you stress because you don't know what you have to look for. What is normal or not. In the first days I had stress to observe it, not knowing if it was normal*.” (M7)
		To be acknowledged in their parenting role	“*To feel supported. I think to feel supported is the most important. That we are valued*.” (F14)
	3.2.3.2 Returning home	Preparation of discharge	“*It's exactly that (discussing about how i twill ne at home), it's missing. We talk right here, right now but not about after discharge*.” (M14)

#### Mothers' needs

##### Experiencing postpartum changes

Giving birth and breastfeeding initiation induced many physical and psychological changes commonly seen after birth. These physical changes frequently induced sleep deprivation and pain. Mothers reported their needs for rest and their pain to be relieved were difficult to meet, as they had to care for their newborn and be active.

“*I had problems to go to the toilets due to (perineal) tear. For me it was really problematic [...]the biggest difficulty for me was 'this body'.”* (M1)

They expressed various needs regarding breastfeeding. They wanted to have information on how to proceed (e.g., breastfeeding position), what it may imply for their body (e.g., sore nipples) and for their relationship with their newborn.

“*I had a lot of questions regarding breastfeeding in fact. Because it was my first time and I didn't know exactly what to do*.” (M14)

They also needed that HCPs recognized and acknowledged the normality of the particular mental state they were in.

“*I'm telling you the first 48 h, just after the birth, […] I wasn't really there, I didn't realize. And then so I don't know, it was quite strange.”(M5)*

##### Emotional needs

After birth, mothers experienced an emotional roller coaster, including joy, fear, and sadness. They needed HCPs to acknowledge their emotions by reassuring them as often as required. Mothers reported being supported emotionally was important. They mentioned HCPs' caring, active listening, and use of reassuring words had a comforting effect.

“*It's true that the caring after the violence of childbirth is just ultra-comforting and ultra-necessary. Because you're immersed in something completely unreal […] be surrounded by people who are really caring, who listen. Well, for me, that's really important.” (M12)*

##### Creating a family unit

After birth, mothers stressed the importance of creating a family unit; for this, the partner's presence and having intimacy were essential. Mothers wanted to adjust to their new role together with their partner. One mother staying in a shared room mentioned the difficulty of being without her partner at night:

“*The more difficult thing [is]…the absence of the father during moments when […] we would like to be together, to build it [parental role] together*.” (M6)

One participant underlined the importance of intimacy by creating a bubble with their partner and newborn, where they could enjoy the first moments of their newborn's life together, despite the presence of HCPs or other people in the room.

“*In terms of support and help... I would have needed to be alone with my partner and our daughter for a few moments.” (M4)*

##### Self-esteem

Mothers needed to feel confident in their new mothering role. Doing things by themselves helped mothers to develop their skills and confidence in their capacity to take care of their newborn.

“*What helps for sure is to have your little one around the clock. In fact, you have no choice… but to be a parent at that time, because the child is there […]. Even if it's hard at first, it helps.” (M 9)*

Some mothers needed to share their experience with other parents, in order to become more confident in their parenting role.

“*Being with someone in the room, another mother, I found it helped because we had a live experience. Even if we're not going to compare ourselves, but the fact of having, next to you, a woman who is going through the same thing, […] allowed me to put some of my situations into perspective.” (M 12)*

##### Difficulty in communicating their needs

Some mothers experienced difficulties to communicate their needs to HCPs. This difficulty was related to their personality traits or values, such as not daring/wanting to disturb HCPs. Their expectations and preconceptions regarding the postpartum hospital stay also induced some difficulties in communicating their needs. For instance, they expected help to come automatically, without asking. Some mothers considered that HCPs should anticipate their needs and offer support accordingly. One mother reported not being at ease about asking for help, as she considered the hospital context to be highly hierarchical.

“*I had the impression that it was very much expected that it was the patient who would say what he/she wanted. […]in this medical context it's hard to speak? […] indeed there's so much of hierarchy... it's not easy.”* (M6)

##### Postpartum stay

This theme includes specific needs related to the hospital context and the ward's organization. Mothers expressed a need for continuity of care. They experienced a loss of reference points due to staff rotation, which was accentuated by their inexperience in their new parenting role. They mentioned not knowing whom to trust. Another need was to receive coherent information. When mothers had incoherent information, they felt unsettled. It also felt complicated to justify decisions that had been agreed with another HCP. This is illustrated in the following verbatim.

“*The teams rotate a lot, which I understand, but in fact, we don't know who to trust, we don't know who has what information, I realized several times that when I rang the bell and asked for something, …people didn't know my medical records.” (M3)*

#### Fathers' needs

##### To be integrated during the postpartum stay

Fathers in our study highlighted fundamental issues for which they required consideration and investment from HCPs. Father reported that the newborn's arrival had impacted in many ways, such as lack of sleep, hunger, and intense emotional experiences. As mothers, fathers also needed to be able to rest, underlining the importance of having a “proper” bed. They also mentioned feeling overwhelmed by the information they were given. They suggested that waiting a little while before receiving information or being able to ask for the same information again would be helpful.

“*We hadn't slept for hours, both of us, and I had fallen asleep a bit during the period before the birth on a chair. It's not a real restorative night sleep […] when you haven't slept for 36 h and you're coming out of something emotionally important your brain may not be at 100%.”* (F17)

Fathers needed to be present at all times with their partner and newborn. Fathers said they did not want to be treated as a “spare part” but rather to be integrated and involved into the postpartum care. If not, they reported worries regarding the separation with their partner and newborn.

“*I think that the most intense moment, it's precisely that night when the baby cries a lot and when, he needs to breastfeed and at the same time, [while] he's breastfeeding, he doesn't eat, but... when he's not there he cries. Well, the fact that I couldn't be there at that moment, it's true that was difficult, with the idea of forming a team with the mother*.” (F6)

##### To be reassured

Fathers needed to be reassured and informed on the health of their partner and their newborn, especially if they could not stay with them. Fathers reported concerns regarding breastfeeding. They also mentioned as essential that HCPs acknowledged verbally that everything was and will be going well.

“*Personally, I would have liked someone to reassure us about breastfeeding and going home, because what I realized was that my wife had problems with breastfeeding […] We got a lot of different advice […] but nobody told us.... at home, it's going to be fine.” (F15)*

##### To be prepared for the postpartum stay

The postpartum ward operated according to explicit and implicit rules. Knowing these rules helped fathers to have reference points. Fathers had many questions related to their stay e.g., how will the stay take place? for how long? Who will be coming to get me? When will the newborn be bathed? Fathers wanted to know how to act and who to contact if they had questions.

“*I think it's more in terms of explaining the planning, of how it was going to happen[…] during the day. […] it's about the time, once they've left,... we don't actually know when they're going to come back.” (F1)*

##### To consider their needs as less important than their partners'

Fathers considered themselves as non-priority for getting support. To the contrary, they underlined their role to support their partner and wanted HCPs to help them in doing so.

“*No, finally, I didn't really need support. I wasn't the one who gave birth. […] What could I need in terms of support? It's good news, it's a birth, it's a positive thing. […] I don't need any support myself […] I was just a mental and physical support, that's all.” (F13)*

Fathers' personality traits also hindered them to ask for support (i.e., they did not dare to ask for support thinking they will bother HCPs). Finally, their expectations and preconceptions regarding hospital care provision also influenced their needs.

“*I had the idea, that someone would look after our daughter […]. I don't know why I imagined that we would arrive in the room and that someone would be able to take care of the little one, so that my wife could rest.” (F10)*

#### Shared parental needs

##### To care for their newborn

Following the birth of their child, parents had to discover what parenting was and how to care for their newborn. In this context, they had many questions. Parents often felt inexperienced, not knowing how to proceed with the newborn care. They also felt afraid or even helpless of not doing the right thing and how to respond to their newborn's needs. Therefore, parents needed to be supported in learning how to care for their newborn. This implied that HCPs gave them information *via* various methods. Some parents found it helpful to have short written information leaflets. Others wanted that HCP demonstrated the care and provided the occasion to do the care with them, to be supervised.

“*There was the bath, […]. At that point, it was clear that they were going to explain to us how to do things, how to dress him, how to change him, so these were things that were important to us, because we didn't know.” (F14)*

Parents needed to be verbally praised on the care they were providing for their newborn and have feedback.

“*Maybe to say regularly that we are doing it (taking care of the baby) well. Like, “you're doing great.”(M11)*

Mothers expressed a greater need for informational support regarding potential newborn's health complications, such as failure to thrive and tongue-tie. Being unfamiliar with these complications, they expressed a need to be properly informed and guided on how to care for their newborn in these particular circumstances.

“*When she was born, she had tremors […]. They (HCPs) said ‘you need to monitor the evolution of your newborn's tremor.' Then you are stressed because you don't really know what you should look for. What's normal or not.” M7*

##### Returning home

The need to prepare for the return home was mentioned by both parents. They reported the importance of organizing the hospital discharge, wanting that HCPs discussed with them how it will be at home in order to better anticipate and manage this transition. Parents did not want to be rushed for hospital discharge, to the contrary they wished HCPS take time to recap the recommendations and do a sort of final briefing so that they can feel ready to go home, like one father who said:

“*It's [information] repeated, but it's quite good that it's repeated, when we saw the doctor just before the discharge. He gave us a general briefing on all the things to do and not to do at home and gave us a good feedback on all the recommendations.” (F14)*

## Discussion

This study explored the perceived social support needs of first-time parents in the early postpartum period with an understanding of the specific needs of not only the mothers and the fathers, but also needs shared by both parents.

### Mothers' needs

To be emotionally supported whilst adapting to postpartum physical changes was important to mothers. In line with other studies, mothers in our study expressed the need to have more support and attention for their emotional and physical changes (37, 49, 50). Practical support was key, as they needed help to care for themselves (51).

Receiving breastfeeding support was essential. In particular, mothers had questions regarding breastfeeding positions and how to proceed. Another study reported that breastfeeding was a main concern for first-time mothers, in particular regarding techniques (52).

We also found that mothers experienced mixed emotions following the birth of their newborn; they needed that HCPs acknowledged these emotions and reassured them. In their study, Mcleish et al. (53) found that mothers experienced the same needs. They wanted that someone would ask them how they felt (54). As in our study, mothers reported feeling comforted by HCPs by using similar terms of being sweet, such as kindness and sensitive staff (51).

Having privacy and their partner's presence was essential for mothers in order to create a family. Mothers mentioned the importance of having a single room, which was also appreciated in another study reporting higher care satisfaction when in a single room (55). Mothers in our study reported the partner's presence as important to co-construct their parenting together. This is similar to other studies, where mothers wanted to rely on their partner for practical help and reported feeling distressed when their partner had to leave, sometimes to the extent that mothers wanted to shorten their stay (51, 53).

Caring for their newborn by themselves was important to mothers, as it gave them confidence. This was also reported in other studies (53, 54). Mothers thought that having the opportunity to practice caring for their newborn empowered them (54). HCP who facilitated this helped to affirm their parental competencies (53).

Mothers' difficulties to communicate their needs was also found in other studies (41, 53, 54, 56). As everything is new to first-time mothers, they do not really know what to ask for (53). Mothers, like in our study, had some expectations regarding their postpartum stay which may have induced this difficulty. Two studies reported that mothers do not ask for support because they think it is the HCPs' professional duty to do so and implicitly assume that HCPs will provide it without asking (41, 56). Mothers did not ask or select what they wanted to ask for support, because they either did not want to bother HCPs or thought their questions might be stupid (53, 54).

This study highlighted the importance of continuity of care. Mothers in other studies explained dissatisfaction about being taken care of by too many midwives, which resulted in inadequate transmission of information and poor continuity of care (49, 56). In our study, mothers also faced difficulties when they received incoherent information. In other studies, mothers also reported negative feelings, such as frustration, when they were confronted with inconsistent advice or when they needed to repeat themselves (57, 58). This sometimes led to a lack of trust in HCPs (53).

### Fathers' needs

Fathers wanted to be integrated into postpartum care during their stay, and wanted HCPs to recognize that, not only mothers but also fathers, feel exhausted, hungry, and overwhelmed with emotions. Other studies found that fathers were experiencing various physical needs and emotional needs due to a loss of sense of reality (2, 25, 43, 59). Fathers wanted to be present for their partner and newborn. Like in other studies, fathers reported worries due to the separation when they were not able to stay overnight (25, 59). Fathers in our study wanted to be included in the care of their newborn. Other studies also showed that fathers reported the importance of being included as a co-parent and to create a bond with their child (2, 59). Fathers wanted to be reassured about what will happen once at home, especially regarding their newborn's health and breastfeeding. Another study found breastfeeding as a main concern for both parents in the early postpartum (52).

Fathers needed to anticipate their postpartum stay, by having information on how the stay would unfold and who they should ask if they had questions. This was also reported by other fathers mentioning the need to clearly know who does what (2, 57) and to have information regarding visiting hours or rooming in (43).

In our study, fathers considered themselves as a support for their partner. Like in other studies, fathers considered their partners' needs as more important than their own (2, 25, 59). This was corroborated by another study where fathers did not tend to focus on their own emotional needs because they thought their partner needed more emotional support than themselves (2). This phenomenon can be explained by the fact that fathers experience an inner conflict between being more involved and not wanting to take the support away from their partner (25, 60, 61). Some fathers report that asking support is socially unacceptable (25). This highlights the traditional and cultural norms of men as fathers and does not necessarily mean that they do not really need support. Indeed, studies in the neonatal context show that fathers tend to hide their own worries and have difficulties talking about feelings and requesting support (62).

### Shared parental needs

Shared themes were “to care for their newborn” and “returning home.” Both parents in our study expressed the need to learn how to care for their newborn. This need has been widely documented in postpartum research for both first-time parents (2, 52, 58). Mothers in our study mentioned specific needs related to newborn potential complications. Particularly, they needed in-depth information in the event of complications (51). This could be explained by the fact that mothers felt responsible for the life of their newborn (58).

To prepare for discharge was a need for both parents. Being able to picture themselves in the future was important. Another study reported that it is essential for parents to be prepared to discharge (57).

Although mothers and fathers had shared needs, most of their needs differed. This gender difference has been found in similar context (63–65). One study reported that parental needs are similar but expressed in a different way (65). This is probably due to social norms of masculinity in Western societies, where men tend not to communicate their needs to others (60, 66). Other explanations may be the primary focus of postpartum care (mother and child) and the views HCPs have of fathers, leading to inequalities in care provision (59, 67). Different parental expectations may explain this difference (68). Indeed, anticipated needs of information was stronger for fathers than for mothers (68). In our study, fathers mentioned receiving information as important. Other studies suggest that fathers' informational need is greater than mothers (30, 60)_._ This may be due to fathers favoring problem-focused coping approaches (60, 61).

### Strengths and limitations

Strengths of this study include the novelty of exploring the perceived formal social support needs of both first-time parents during the early postpartum period. The sample in our study is representative of the local population. Indeed, mode of birth of the participants in this study is comparable to national and international rates: 61 vs. 55% of spontaneous birth and 22% of cesarean section vs. 33% in 2016 in Switzerland (69).

Trustworthiness of the study was enhanced by the utilization of triangulation, peer-review and debriefing related to data analyses. Researchers assured the study's credibility by presenting the results to participants and asking for their feedback (70).

This study has some limitations, even if researchers tried to equally involve fathers, more mothers participated; nevertheless, we were able to reach saturation for fathers. Providing feedback for fathers was not possible, as none of the fathers engaged with this process. This is in line with other studies conducted in the perinatal context (7, 71). This may be because fathers, due to shorter paternal leave, do not have the time to participate or feel less concerned by postpartum research (72). Underrepresented or marginalized populations, such a single-parent families, migrant parents or those whose newborns were at the NICU were not included in our study, so our results do not necessarily apply to this population who may have other specific needs. This study did not include any other considered vulnerable time periods in the transition to parenthood that may be considered as vulnerable, as the early postpartum period was identified as critical and lacked evidence for practice. Furthermore, no data was collected on previous mental health problems as risk factors for PMAD.

### Clinical implications and suggestions for future research

Overall, in our study parents had a positive experience of their stay. However, some parents experienced difficulties related to incoherent information, lack of information on their stay, and lack of preparation for discharge. Fathers particularly experienced mixed feelings regarding their integration in the care, even though this study did not specifically focus on the partners' satisfaction. When reporting their experiences and needs, parents gave insight into their social support needs but also suggestions for improving their care. Parents' suggestions were related to HCPs being available and attentive to their needs and sufficiently staffed. HCPs should integrate fathers during the stay by considering their own physical needs and offering them the possibility to organize and participate in, if possible, the newborn care when they are present. HCPs should also be conscious of their personal beliefs and cultural norms that may shape their interactions with fathers. Further research should be conducted with more vulnerable populations, such as migrant, single-parent families and parents whose newborn is in the NICU.

## Conclusion

The results of our study provide new knowledge and understanding of both first-time parents' perceived formal social support needs during the early postpartum period. First-time mothers and fathers had specific but also shared needs. Mothers wanted more emotional support than fathers. Fathers mainly wanted to be included more in the care. HCPs should strive to better include fathers during the postpartum stay.

## Data availability statement

The datasets presented in this article are not readily available because individual data set generated during the study is not publicly available, due to concerns of potential violations of participants' privacy. However, the final data set (in French) for analysis is available. Requests to access the datasets should be directed to antje.horsch@chuv.ch.

## Ethics statement

The studies involving human participants were reviewed and approved by the Ethics Committee for Research on Humans of the Canton of Vaud, Switzerland. The patients/participants provided their written informed consent to participate in this study. Written informed consent was obtained from the individual(s) for the publication of any potentially identifiable images or data included in this article.

## Author contributions

ES contributed to conception and design, participants' recruitment, data collection, transcription, analysis, and manuscript writing. MV participated in data analysis and manuscript writing. A-SR and AH contributed to the conception and design of the study, participated in discussions related to data analysis and manuscript review, provided critical feedback, and supervised the work of ES (Ph.D. candidate) and MV. A-SR and AH gave final approval of the manuscript's publication. All authors have read and approved the final manuscript.
