# Erratum: Social support needs of first-time parents in the early-postpartum period: A qualitative study

**DOI:** 10.3389/fpsyt.2023.1171192

**Published:** 2023-03-03

**Authors:** Frontiers Production Office

**Affiliations:** Frontiers Media SA, Lausanne, Switzerland

**Keywords:** social support, early postpartum, mothers, fathers, needs, qualitative

An omission to the funding section of the original article was made in error. The following sentence has been added: “Open access funding was provided by the University of Lausanne.”

The original article has been updated.

